# Underestimating attacks: comparing two sources of publicly-available data about attacks on health care in 2017

**DOI:** 10.1186/s13031-023-00498-w

**Published:** 2023-01-30

**Authors:** Vanessa Parada, Larissa Fast, Carolyn Briody, Christina Wille, Rudi Coninx

**Affiliations:** 1 Helpcode, Genoa, Italy; 2 HCRI, University of Manchester, Manchester, UK; 3 Intelligence Innovation and Integration Unit, Health Emergency Intelligence and Surveillance Systems Division, WHO, Geneva, Switzerland; 4 Insecurity Insight, Vevey, Switzerland; 5 Health Emergencies Programme, Interagency Policy for Emergencies Unit, WHO, Geneva, Switzerland

**Keywords:** Attacks on health care, Conflict, Health, Conflict event data, Data quality, Publicly-available data, Media data

## Abstract

**Background:**

Attacks on health care represent an area of growing international concern. Publicly available data are important in documenting attacks, and are often the only easily accessible data source. Data collection processes about attacks on health and their implications have received little attention, despite the fact that datasets and their collection processes may result in differing numbers. Comparing two separate datasets compiled using publicly-available data revealed minimal overlap. This article aims to explain the reasons for the lack of overlap, to better understand the gaps and their implications.

**Methods:**

We compared the data collection processes for datasets comprised of publicly-reported attacks on health care from the World Health Organization (WHO) and Insecurity Insight’s Security in Numbers Database (SiND). We compared each individual event to compile a comparable dataset and identify unique and matched events in order to determine the overlap between them. We report descriptive statistics for this comparison.

**Results:**

We identified a common dataset of 287 events from 2017, of which only 33 appeared in both datasets, resulting in a mere 12.9% (n = 254) overlap. Events affecting personnel and facilities appeared most often in both, and 22 of 31 countries lacked any overlap between datasets.

**Conclusions:**

We conclude that the minimal overlap suggests significant underreporting of attacks on health care, and furthermore, that dataset definitions and parameters affect data collection. Source variation appears to best explain the discrepancies and closer comparison of the collection processes reveal weaknesses of both automated and manual data collection that rely on hidden curation processes. To generate more accurate datasets compiled from public sources requires systematic work to translate definitions into effective online search mechanisms to better capture the full range of events, and to increase the diversity of languages and local sources to better capture events across geographies.

## Background

Attacks on health care facilities, medical transport, patients, and personnel occur too often around the world, in both conflict and non-conflict situations [eg 1–5], with more than 800 reported incidents in 2021 [6]. Research about the scope and scale of attacks [7] has pointed to particular cases where attacks have generated significant attention, such as the Syrian [eg 8–11] or Ukraine [eg 12–14] conflicts and to increased numbers of reported attacks of violence or obstruction of health care more generally [6, 15]. Not only do such attacks destroy vital human and health resources, in many cases they violate International Humanitarian Law (IHL) [16–18]. They deprive people of urgently-needed care, undermine the health system, hinder public health services, including health-related Sustainable Development Goals, and cause detrimental social and economic consequences.

In 2016, the United Nations Security Council passed Resolution 2286 condemning attacks on health services in situations of armed conflict. The International Committee of the Red Cross (ICRC) and Médecins Sans Frontières (MSF) each launched initiatives to raise awareness and document violent attacks on health care, via the ICRC’s 2011 sixteen-country study [1] and its Health Care in Danger initiative [19], and MSF’s Medical Care Under Fire campaign [20]^1^ and related research [eg 21, 22]. In 2012, the World Health Assembly adopted Resolution 65.20, tasking the World Health Organization (WHO) with leading the development of methods for systematically collecting and disseminating data about attacks on health facilities, workers, vehicles, and patients in complex humanitarian emergencies.

Implementing Resolution 65.20, in 2014, WHO started to collect publicly-available, open source reports of attacks, and published these data on its website to document and quantify the magnitude of the problem of attacks on health care [23]. In the meantime, WHO developed a more robust methodology [24] to collect data on attacks on health care, and in December 2017, launched the Surveillance System on Attacks on Health Care (SSA), which collects and confirms primary data from approximately 14 countries and territories [25]. In these locations, WHO field offices collect information via their partners on the ground and input incidents directly into the SSA database.

At the same time, the Swiss non-profit organisation Insecurity Insight was tracking events that interfere with the delivery of aid, including attacks on health care, via its Security in Numbers Database (SiND), an initiative of the Aid in Danger project [26]. Insecurity Insight collects and collates public data from media reports, social media, and other publicly-available sources as well as from field-reported, often confidential data from its approximately 30 non-governmental organisation (NGO) partners that directly submit incident reports for inclusion in the Aid in Danger project datasets. For health care, Insecurity Insight collaborates with other organisations under the Safeguarding Health in Conflict Coalition (SHCC) umbrella to produce annual reports that document attacks on health care globally, using open source media reports, other publicly-available data as well as confidential data from Insecurity Insight and other SHCC members [6, 15]. Together the WHO and SHCC efforts represent the most comprehensive and publicly accessible multi-country data sources about violent attacks on health care currently available for emergency and armed conflict contexts.^2^

**Fig. 1:**
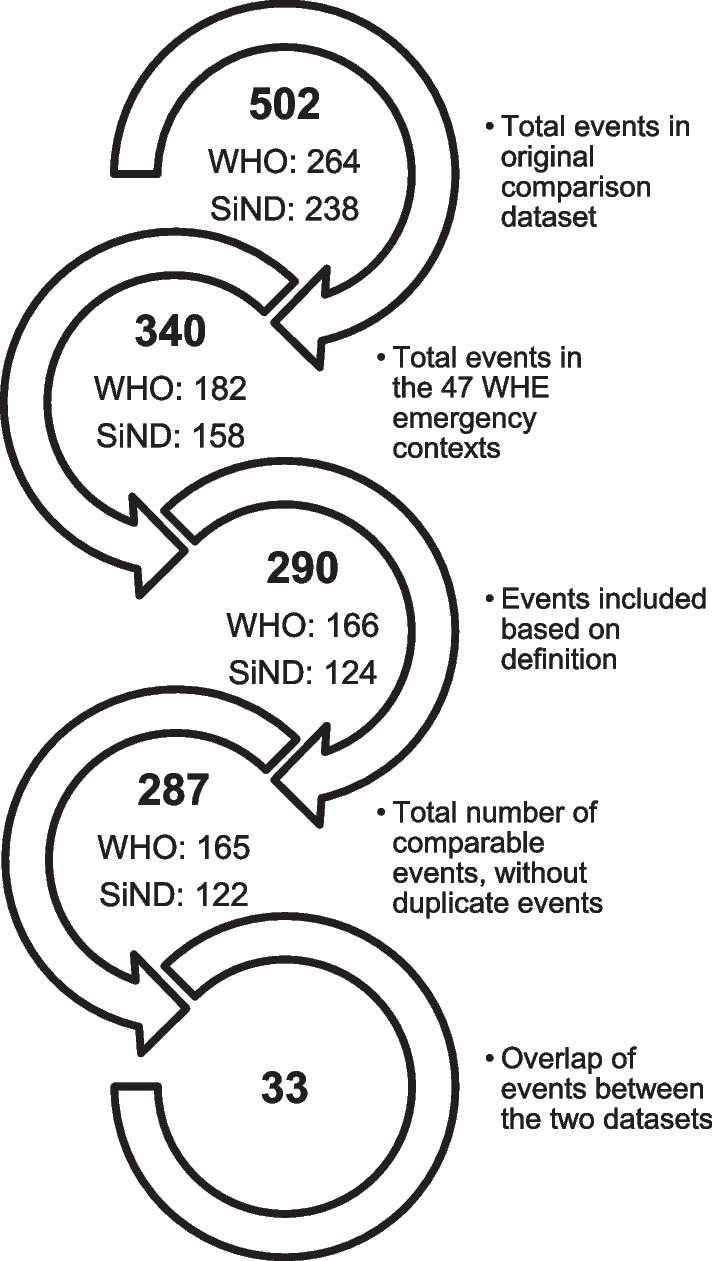
Data preparation process with summary of event data and inclusion filters

This article originates from efforts to compile the 2017 SHCC report, which presented us with a puzzle. As reported below, in the process of cleaning and collating the publicly-available WHO and Insecurity Insight SiND health care data, we discovered a similar number of events overall for selected countries (264 and 238 respectively, see Fig. 1) yet our analysis revealed only minimal overlap (12.9%) between datasets.^3^ What accounts for this unexpected result? And what does it tell us about the number of attacks against health care more generally?

These questions remain relevant, even several years later. Experts generally recognise that attacks on health care are underreported and under-analysed [1, 6, 7, 27]. Furthermore, research about the use of media reports in analyses of social phenomenon, such as violent events [28, 29] and collective action [30], suggests that publicly available information contains selection biases (related to the issue, the location of the event, and the reporting agency) and issues pertaining to the veracity of reporting (e.g., about the ‘hard facts’ of location, date, casualty, and actors) [eg 29, 31, 32]. For instance, Weidmann [29] examined the veracity issue through a comparison of conflict events in Afghanistan reported through the media and through a military dataset. For casualty data, Weidmann’s two datasets corresponded in approximately half the events. Where they differed, media data tended to report slightly higher casualty data, with differences within ‘reasonable limits’. Other analyses of data sources about state repression point to the importance of a diversity of sources in minimising bias [31]. In analysing civilian harms and violence in Guatemala, Davenport and Ball [33, p 428] found: ‘Within our investigation, newspapers tend to focus on urban environments and disappearances; human rights organizations highlight events in which large numbers of individuals were killed and when large numbers were being killed throughout the country in general; and finally, interviews tend to highlight rural activity, perpetrators, and disappearances as well as those events that occurred most recently’.

These studies and our original conundrum illustrate the need for deeper investigation into data about attacks on health care, their sources, and their implications. This includes the type of data and their public or confidential nature, the criteria for inclusion, as well as the flow of reporting and verification of information that characterise data collection processes. For example, social media posts are frequently published minutes after attacks. Local and international media stories follow, sometimes hours later. These stories are updated as more information becomes available, potentially leading to conflicting and duplicate data about victims, perpetrators, or circumstances, depending on the original source and which version of the updated story is used. Moreover, the language of the original story or post and the language capabilities of those compiling public data influences their inclusion, as do the invisible algorithms that determine which news stories appear in internet searches.

Field-based accounts represent another important source of data, reported by organisations affected by the attacks, by individuals who witness attacks or share testimony with journalists, or by humanitarian or other organisations that collate this information. Many data collection efforts for attacks on health care have a specific geographic or contextual focus, such as for Syria [8–11], Yemen [34, 35] or Myanmar [3], or in emergency contexts (such as the current WHO SSA) or in armed conflict contexts [1, 7, 36]. In many cases, these efforts rely on field-based data collection in addition to publicly-available data. In compiling global datasets, the intensive focus on one or a few countries can lead to over-representation for particular countries^4^ and create inadvertent gaps in a more holistic understanding of the issue. The challenges of gathering accurate field-based data in situations of conflict are often significant and well-documented [eg 29, 36–38]. While field-based data are often seen as more reliable, issues of access, trust, and existing social networks influence information flows and therefore which attacks are reported (or not reported), to whom, and therefore whose stories are told. This affects available data for analysis, with implications for our understanding. In the case of attacks on health care, ensuring the continued security of operational activities dictates a need for restricted data sharing, which may mean that key details such as location are missing from public accounts. This, in turn, hinders efforts to avoid double counting events when compiling data from different sources.

The need for better understanding of these issues is pertinent, as publicly-available data are particularly important in documenting attacks on health care. In some cases, publicly-available data are the only regularly accessible source of data about attacks; where field-based data are available, they constitute a complementary data source. Advocacy efforts, such as the SHCC, are dependent upon public information to raise awareness and maintain attention on these issues, highlighting the relationship between advocacy and data gathering [5, 21, 39, 40]. Thus, even where field-based data exist, publicly-available data will continue to be used in awareness-raising campaigns and to support policy discussions informed by specific incidents and attack patterns. Moreover, the reason for collecting data influences the type, detail, and uses of data. Aside from the mandate bestowed on WHO by the WHA, organisations gather data for advocacy, investigation and accountability, operational security and protection, or research purposes [5, 34, 37, 38, 40–42].

As a result, further investigation is needed into data collection methodologies, including the processes used to identify and collate incidents of attacks, the extent of underreporting, and the implications for our understanding of and responses to attacks on health care. Up to now, issues of data collection processes and their implications have received little attention, despite the fact that these processes generate confusion when they result in differing numbers [5, 38, 39, 43].

This article aims to contribute to understanding these gaps by comparing the data collection processes of two publicly-available datasets derived primarily from traditional media sources. While the hidden biases of field-based data collection deserve further investigation, as does the importance of social media reporting, which has increased since 2017, these topics are not the focus of this article. Instead, given the importance of public data for both documenting and raising awareness about attacks on health care, and with relevance for research and advocacy purposes, this article aims to investigate and compare the data collection processes and issues of bias in publicly-available data about attacks on health care. In doing so, it makes two important contributions: (1) it provides insight into the extent of under-reporting of attacks on health care more generally; and, (2) it highlights some of the less visible types of bias present in publicly-available datasets about attacks on health care. To better understand the contributions and limitations of using publicly-available data, we compared the WHO and Insecurity Insight datasets of publicly-reported attacks on health care for the year 2017.

## Methods

### Definitions

Both the WHO (from 2014 onwards) and Insecurity Insight (since 2011) regularly collect and code publicly-available information and data shared by field organisations^5^ about attacks on health care using an event-based approach.^6^ The WHO attacks data focus only on health care, defining an attack as ‘any act of verbal or physical violence or obstruction or threat of violence that interferes with the availability, access and delivery of curative and/or preventive health services during emergencies [2]. In 2016–2017, prior to the development of the SSA, WHO surveillance concentrated on countries and territories facing acute or protracted emergencies with health consequences resulting from any hazard, all of which fell under the WHO Health Emergencies Programme [45].

The SiND adopts a broader approach to data collection that encompasses incidents that negatively affect staff, infrastructure or the ability to deliver health, aid, education, or protection. The health dataset therefore constitutes one part of the overall SiND. The health dataset uses the definition of ‘an incident that negatively affects staff, infrastructure and/or the ability to deliver health care’ to determine inclusion [46]. While the principal focus is on ‘events’, the SiND allows for continuous events, which capture processes such as administrative impediments (eg visa or import restrictions) or laws (eg those that preclude foreign funding) as long as they negatively impact on or obstruct health care provision. Additionally, SiND events are not geographically limited, thus covering more than the 47 WHO priority countries. Definitional variance from the 2017 WHO definition meant the SiND encompassed protest events that interfered with health service accessibility, availability or delivery.

Both datasets, however, included events that negatively affected local heath structures supported by ministries of health or private health care providers, as well as intimidation, threat, or physical violence against health workers by patients, their families, other civilians, states, and non-state actors.

### Data sources and collection processes

Although both organisations’ data collection methodologies have evolved over time (including as a result of this analysis), we report here on the sources and processes used to create the datasets analysed in this article in an effort to gain insight into the biases of publicly-available data and the extent of missing data. We chose 2017, as it represented a middle stage when data collection processes for both organisations were already well established, yet before the WHO’s shift to the SSA methodology in 2018. Moreover, these were the only two datasets available at the time that focused on attacks on health care in multiple countries, and that clearly identified recorded incidents from publicly-available sources for which it would be possible to compare individual records. The datasets used in this analysis similarly comprise publicly-available data only. Crucially, as we report below, both initiatives involved extensive efforts to collect media and other open-source reports, yet they differed in terms of the actual sources consulted and the processes (automated searches involving keywords vs manual searches in relevant sources) used to detect events. In articulating these processes in more detail, we identify some of the biases inherent but often invisible in the processes of identifying events from publicly-available data, and also examine the extent of underreporting in these data sources.

To create its 2017 dataset, WHO reviewed daily *automated* Google Alerts comprised of English-language keyword searches^7^ and systematically examined a set of additional sources.^8^ The reported incidents included only individually-reported events and not aggregated figures. For example, reports describing several attacks within a period of time or in a region but without details of specific attacks were not included. WHO data were compiled in a WHO-developed software to provide data in humanitarian emergency settings, and then cleaned and analysed in Excel.

To compile its 2017 dataset, the SiND collated publicly-available data through regular, manual reviews of English-language mainstream news outlets, humanitarian-focused news sources, and curated newsletters focused on aid, violence, and development, including one that provided English translations for local news sources.^9^ Thus, instead of keyword searches, the SiND process employed *manual* searches of news articles to identify events from among a set of generalist and specialist news outlets, including those specifically geared toward emergency and conflict response.

Both the WHO and SiND 2017 datasets were coded by country, type of event, and immediate impact on health workers or facilities (e.g., referring to death or injury to health workers, damage to or destruction of facilities) and, in the case of the SiND, the impact on health care delivery (e.g., denial of access to health care services, where violence or fear prevent patients from accessing services, or violence and insecurity prevent health workers from providing these services). Both included the date of the attack, the country, sub-national location (where available), a description of the attack, the type of attack, health care resources affected, the number of victims, and, where available, the perpetrator.

### Study design and methods

Comparing the datasets involved a two-stage process: first, to ensure comparability by assessing whether individual events met our inclusion criteria, and second, to determine the overlap between datasets, including efforts to determine the extent of underreporting. We used a basic event record comparison (based on record-linkage from the Multiple Systems Estimation or capture/recapture methodology [48]) to determine overlap between the datasets and the extent of under-reporting, as well as a more detailed event comparison to identify patterns in the nature, source, or type of overlap. Even though our data did not meet the strict conditions for a capture-recapture analysis [48], we conducted this analysis to determine a crude estimate of the possible extent of underreporting. Using the Chapman estimate [49] as a conservative parameter, we used the equation N = (r1 + 1)(r2 + 1)/ r12-1 -1, where r1 is the number of events in the first dataset and r2 references the second dataset, and r12 is the number of events captured in both datasets, to arrive at an estimate of for the minimum number of events in these 31 countries ($\frac{(165+1)(122+1)}{33-1}-1=x$). These findings are reported in the Results section.

To prepare the datasets for analysis, we first eliminated from our analysis all events outside of the WHE framework (see Table 1) and in the Syrian Arab Republic. The WHE framework designates WHO responses in grade 1–3 emergencies as well as ‘non-graded protracted emergencies’, with grade 3 referring to the most acute emergencies in the 2016 update—the latest date available for our 2017 data. Thirty-one of these were emergency contexts, with an additional 16 protracted crises. Although WHO surveillance concentrated on the countries in Table 1, the WHO data did include events outside these countries. To maximise our dataset, our analysis encompassed all 47 countries in Table 1 except Syria. Syria events comprised a significant proportion of all available data for WHO and the SiND, but due to incomplete or missing information it was impossible to eliminate double-counted events or to ensure accuracy in matching event records.

**Table 1:** WHE Grade 1–3 emergencies and protracted emergencies (from 2016) [45]

WHO Grade 3 Emergencies	WHO Grade 2 Emergencies	WHO Grade 1 Emergencies	Non-graded Protracted Emergencies
Iraq Nigeria South Sudan Syrian Arab Republic Yemen	Angola Cameroon Central African Republic (CAR) Democratic Republic of the Congo (DRC) Ecuador Ethiopia Haiti Libya Myanmar Niger Ukraine United Republic of Tanzania	Afghanistan Bangladesh Democratic People's Republic of Korea (DPRK) Fiji Indonesia Kenya Mali Nepal Pakistan Papua New Guinea Sri Lanka Thailand The Philippines West Bank and Gaza Strip	Burkina Faso Chad Colombia Djibouti Egypt Guatemala Honduras Jordan Lebanon Mauritania Senegal Somalia Sudan The Gambia Turkey Zimbabwe

Next, two coders separately examined each individual event by dataset (WHO or SiND) to determine whether the event matched our inclusion criteria. The inclusion criteria required that an event met the WHO definition of attack (defined as ‘any act of verbal or physical violence or obstruction or threat of violence that interferes with the availability, access and delivery of curative and/or preventive health services during emergencies’) and that occurred within one of the 47 WHE programme countries for 2016.

Both coders also separately defined a match status for each individual event (see Table 2; see also [9] for another study using this methodology). When an event appeared in both datasets, each coder identified the ID number of the corresponding event in the other dataset based on location and identifying information (location, date of the attack, name or type of facility, name or affiliation of victim(s), and perpetrator type). We then compared our results (see Fig﻿. 1). Where coding discrepancies existed (either based on inclusion criteria or defined as a ‘possible match’), we discussed the event in question and reached a consensus about inclusion and match status, moving all ‘possible matches’ to either the definite match or unique event category. This process identified three duplicate events, out of 290 events, which we excluded from the final analysis. In several cases, we conducted additional web searches based on the event descriptions in order to determine match status.

**Table 2:** Basis for determining match status

Match status categorisations	
Definite match	Events for which two or more of the following matched: (1) the date of the attack, (2) location or (3) the name of the facility or of the victim
Unique event	Events for which only one or none of the following matched: (1) the date of the attack, (2) location or (3) the name of the facility or of the victim
Possible match	Events for which one or more of the following appeared to match: (1) the date of the attack, (2) location or (3) the name of the facility or of the victim For events in this category, the coders discussed and reached consensus, moving all possible match events to either the unique or definite match category

We report the findings of our comparison with descriptive statistics in our Results section below (see Data Comparison).

## Results

In our effort to compile a comparable list of attacks on health care events, we found that both WHO and Insecurity Insight recorded a similar number of events in the comparison datasets^10^ (264 and 238 respectively before applying our filters, and 165 and 122 afterwards, as reported in Fig. 1). Despite this similarity, our detailed analysis revealed only minimal overlap (33 events, 12.9% of total events) between datasets.

As Fig. 1 illustrates, in preparing our data, excluding events based on the 47 WHE countries decreased our available data by approximately 80 events per dataset. Furthermore, the coding process identified 34 SiND events and 16 WHO events that did not meet the definition criteria and were excluded from the analysis. For comparability, we used the WHO definition of an attack, which resulted in the exclusion of more SiND events in the final dataset. For example, although the SiND had 5 events in Angola, all were protest events that did not match the inclusion criteria. The same occurred in Kenya, where the SiND captured two events, neither of which met the definition inclusion criteria.

In general, the SiND captured data for more countries than did the WHO dataset (see Table 3), likely a function of the global purview of the SiND. Nevertheless, neither dataset identified events for all 47 countries in the 2016 WHE list (see Table 1). Instead, together the WHO and SiND data collection processes found data on attacks against health care in only 33 of the 47 WHE emergency contexts. In two of these countries (Angola and Kenya, as mentioned above), the SiND data were excluded because they did not meet the inclusion criteria, and the WHO data did not capture any events for these two countries, taking the total number of countries/territories in the sample to 31.

**Table 3:** Comparison of datasets, summary of country data

	WHO	SiND
Number of countries/territories with reported attacks on health care (not including Syria)	36	56
Number of countries/territories with reported attacks that were *excluded, *as falling outside the 47 WHE emergency contexts	13	25
Number of *additional* countries/territories excluded after definition inclusion criteria applied	1	6
Total countries/territories with events included in final dataset	23	26

Both datasets skew toward a select number of countries, although WHO appeared to capture more events on average per country than did the SiND (5 vs 3 respectively). The WHO data included more than five attacks in 2017 in a majority of countries (14/23), whereas the SiND reported more than five attacks in only nine of 26 countries. These proportions decrease to 8/23 for WHO and 4/26 countries for the SiND where reporting more than 10 attacks. Neither dataset captured events in DPRK, Djibouti, Ecuador, Fiji, Guatemala, Haiti, Honduras, Indonesia, Mauritania, Myanmar, Senegal, Sri Lanka, or Tanzania.

From the 287 total events (prior to matching) the coders identified and discussed 16 possible matches in order to reach consensus, eventually categorising four as definite matches and 12 as unique events. This left a total of 33 events common to both datasets and an N of 254 events (132 + 89 + 33). This produces an overlap between datasets of only 12.9% (33/254). Furthermore, our basic capture-recapture analysis [48] suggests an estimate of at least 637 events in these 31 countries. This indicates a significant underreporting and the need to identify ways to improve data collection. We address this further in our Discussion section.

### Data comparison

In this section, we compare the datasets, specifically in terms of the type of attack, actors, victims, and geographic location. Attacks on health care were reported in every month, with lows of 15 events in both June and July, and a high of 34 events reported in December. As Table 4 illustrates, the datasets contained significant variation in the type of attack. Events affecting medical personnel appeared most often in the entire dataset, followed by attacks on facilities. Neither dataset recorded a high proportion of attacks affecting patients.

**Table 4:** Type of attack, all events (unique events and definite matches)

Type of attack	WHO (%)	SiND (%)	Total (Matches in parentheses) (%)
Affecting only access to or delivery of health care	2 (1.2%)	29 (23.8%)	31(10.8)
Affecting ambulances	20 (12.1%)	9 (7.4%)	29 (3) (10.1%)
Affecting facilities	47 (28.5%)	25 (20.5%)	72 (9) (25.1%)
Affecting medical personnel	90 (54.5%)	53 (43.4%)	143 (19) (49.8%)
Affecting patients	6 (3.6%)	6 (4.9%)	12 (2) (4.2%)
Total	165 (100%)	122 (100%)	287 (100%)

The WHO dataset reported more incidents affecting ambulances, medical personnel, and facilities. On the other hand, the SiND more consistently captured events related to the denial of access or attacks affecting the delivery of care.

Of our 33 definite match events, 12 (36.4%) affected well-known health care actors in conflict, including 9 (27.3%) specifically affecting MSF or Red Cross societies and personnel. In our overall data, 29 events (13.1%, n = 221) affected MSF or Red Cross societies and personnel. For both unique and definite match events, the numbers of attacks affecting personnel is higher than for infrastructure, whether ambulances or facilities.

Finally, with regard to location, the largest number of definite matches (21.2%, 7/33) occurred in large or capital cities. Within our total dataset, however, 13.9% (40/287, 34 SiND and 6 WHO) of events lacked a specific location. Almost 70% (199/287, 78 SiND and 121 WHO) occurred in urban settings, with only 16.7% in rural contexts (48/287, 10 SiND and 38 WHO). Moreover, definite matches were identified in only nine countries (Afghanistan, CAR, DRC, Iraq, Libya, Nigeria, Pakistan, South Sudan and Sudan).

## Discussion

The discussion above lays the foundation for the two contributions of the study, being first, insight into the extent of underreporting about attacks on health care generally and with public data specifically, and second, issues of bias in public data sources. We discuss each of these in turn. As we discuss further below, the minimal number of matches (33 of 254 events, or 12.9%) suggests not only dramatic underreporting of attacks on health care, but also points to the need for closer examination of public data reporting methodologies to advance efforts at replication. Moreover, it emphasises the need for systematic, comparable data.

Most importantly, the lack of overlap points to substantial underreporting of attacks on health care in public sources. As reported above, a basic capture-recapture analysis [48] suggests the minimum number of events in these 31 countries is at least 637 events. Based on this, the low percentage of overlap suggests that each dataset only captures approximately 45% of the publicly-reported events that occurred in these 31 countries. Both datasets therefore appeared to dramatically underreport the total number of publicly-available data events, and neither search method appeared able to identify even half of public events. Most concerning, given the well-accepted consensus that attacks on health care are underreported [6, 7, 36, 37, 50], this study suggests that the underreporting of attacks is greater than we feared and that the true burden of attacks is significantly higher than current convention allows.

This dearth of overlap between two comparable (based on definition and country/territory) datasets using public information was unexpected, and the characteristics of the data do not entirely explain the low overlap between the two datasets. For example, events captured in 22 of 31 countries (71%) lacked any overlap. On average, WHO captured more events per country than did the SiND, which could be a function of the specific focus on particular countries for WHO as opposed to a global focus for the SiND, and of the adoption of the WHO definition, as noted above. This suggests that geographic coverage likely accounted for some but not all of the variation in datasets.

Given this, what accounts for the lack of overlap? And what do these datasets contribute to our understanding of underreporting and the state of existing data, the process of collecting publicly-available data, and their value for advancing our understanding of attacks and their consequences? We highlight three insights that begin to answer these questions, with a particular focus on issues of bias in the collection of public source data that can help to account for the discrepancies that we found in the two datasets in question. These relate to the subtle yet important influence of dataset parameters and definitions in identifying publicly-available data events, differences in sources, and the need to better understand the actual processes of collecting publicly-available data and their implications. While open source investigations [47], the growing importance of social media for reporting events, and the development of artificial intelligence and machine learning have advanced since 2017 and help to address some of these concerns, we believe the issues highlighted here deserve further reflection. We conclude with some potential ways forward.

First, closer comparison of the object of attack suggests that dataset definitions affect data collection, potentially in subtle ways. As noted in the descriptions of the datasets, the focus of each initiative varied, thus making each more likely to pick up different types of events even when using the same definition of an attack for the purpose of analysis. For example, the SiND accounted better for access events, such as obstructions to health care delivery (n = 29). This is potentially because the Aid in Danger project includes access constraints, bureaucratic impediments, and other events that interfere with the delivery of aid as an explicit focus of Insecurity Insight’s overall data collection effort. Similarly, many SiND-reported attacks did not involve physical violence but instead reported indirect violence, strikes, or document lack of access to health care, such as curfews or the withdrawal of staff because of security threats. By contrast, the WHO compiled information about few such incidents during 2017 (only 2% of events, as indicated on the 2017 dashboard).^11^ Likewise, because neither dataset emphasised nor systematically accounted for attacks on patients, these objects of attack are least apparent in the data despite this being one of five key types of attack [2, 7, 24]. In this way, the database parameters appear to account for some of the variation between the datasets.

Source variation appears to best explain the lack of overlap between the two datasets. In 2017, the SiND did not include any Armed Conflict Location and Event Data (ACLED) project events, whereas the WHO dataset featured a significant number of events from this source (50.3%; n = 83).^12^ This represented the biggest difference between the two dataset sources, and therefore influenced event coverage. ACLED collects data about political violence and protest events from around the world.^13^ The WHO dataset captured political violence resulting in deaths of medical staff and patients, likely stemming from this focus of the ACLED dataset. Importantly, because the SiND disaggregates event types (by attacks on aid, education, health), it is possible that these were included in other SiND datasets yet excluded from the health dataset because the primary object of attack is not health care. While the SiND data included some ACLED reported events in 2017, the dataset did not consistently use ACLED as a source until 2018.^14^ On the other hand, by using information from humanitarian news outlets, the SiND was able to capture events related to access constraints that are a subject of concern for humanitarian actors. These types of events fall outside ACLED and other similar data collection efforts that focus on political violence or protest.

Finally, in an effort to increase transparency, we reflect on the processes of collecting publicly-available data, in particular the differences between automated and manual search processes. Clearly both approaches have weaknesses, since neither approach captured even half of what a capture-recapture analysis suggests as the total number of publicly-available events for our list of 31 countries/territories. Automated searches, such as the Google keyword searches used to capture publicly-available data for WHO, offer more control over the search process through the choice of keywords. Automated searches also broaden reach across the plethora of public sources, since it is possible to identify events from previously-unknown sources. The complexity of terminology, however, can make it difficult to identify and effectively capture events. For instance, determining who are health (or medical) personnel, and what constitutes a health facility can require a level of knowledge or detail that may not be present in a media report. For example, ‘health personnel’ captures a broader range of roles than ‘medical personnel’, and reference to health ‘facilities’ could exclude a mobile health clinic, since facility implies a building of some kind; media sources also often refer to ‘hospitals’ or ‘clinics’ over facilities [41]. Therefore, even with efforts to ensure inclusivity, subtle distinctions in the terminologies used in sources as well as the keyword searches themselves can influence results, particularly in going beyond the more commonly tracked events involving casualties (both death and injury). Narrowing search terms may inadvertently exclude events, just as making searches too broad results in overwhelming amounts of information, much of which is irrelevant.

On the other hand, manual data searches offer the ability to target particular sources, especially those focused on the topic at hand, and to provide more specificity in tailoring a search. However, these searches may create inadvertent biases and entail a smaller reach since they require prior knowledge of a source and repeated, systematic return to all known sources. This requires significant human labour, entailing a trade-off between the labour required to search all relevant sources to identify an additional event [53]. Just as the universe of keywords can make the task overwhelming, so too can the possible number of relevant sources. Curated newsletters represent a way to narrow the sources but introduce additional selection biases that are layered on top of the ones that already manifest in public data more generally.

Crucially, however, both approaches rely on hidden curation of events. For keyword searches, the invisible algorithms of the search engines, whether Google or any other platform, govern data collection and are influenced based on past search history, language, and featured sites [54]. For manual searches, particularly those that rely on curated sources, the searches are influenced first by the choice of which curated source to check, and then by the unknown search criteria and processes of these curated sources. Importantly, we cannot say anything about the selection bias of the original media reports, nor about the curated sources. Yet this highlights our point: that selection biases are multiple, layered, and often hidden. More specifically, they are related to the initial choice of a reporter and news outlet to report particular events, and then later of the (hidden) algorithms used to curate newsletters and searches, whether by major tech companies such as Google, or by the daily news summaries that arrive in people’s inboxes, whether compiled by major news outlets or by humanitarian, peace, or development organisations.

Consulting publicly-available sources alone is clearly not sufficient to capture the extent of attacks on health care [see also 5]. Many events are not of sufficient media interest to be reported, nor do media outlets systematically or consistently document these types of attacks, creating selection bias in the sources themselves [29, 53]. In other cases, such as kidnapping, aid agencies have specifically requested that traditional media outlets not report the event in order to facilitate the safe return of staff members, making these events invisible in public documentation. In other instances, health care providers may prefer not to publicly report armed entries into facilities and instead address such concerns in a diplomatic manner. Consequently they too remain invisible in publicly-available data.^15^

The findings of this study illustrate that while traditional media sources constitute a significant source of information about attacks on health care, these data are dispersed across sources, sites and languages and represent only a fraction of the violence that afflicts health care. Since 2017, both datasets have evolved. WHO created the SSA to document—and verify—attacks on health care in selected emergency contexts, with the assistance and participation of partners and offices in these contexts. Moreover, the WHO team has updated its methodology, using the WHO-led Epidemic Intelligence from Open Sources (EIOS) system to collect public data. The EIOS system analyses the text of articles from over 13,000 sources, covering numerous languages, to determine matches between the article text and defined keyword categories in the system. If the text matches, the article is pulled in the system. The team created attacks on health care as a category, and in 2018 transitioned to using this system for collecting public data.^16^ The SiND has likewise evolved. Insecurity Insight now uses a fully automated process to identify and classify publicly-available articles using natural language processing, which are then manually coded. It has significantly expanded its collaboration with NGO partners and directly collaborates with medical professionals in some countries. It now also systematically incorporates ACLED-reported events. A team of Insecurity Insight researchers continue to systematically review local data sources, including social media, to document attacks.

These improvements can be double-edged, however. Just as growing international attention to this issue makes it more likely that news media outlets report attacks, whether due to high-profile events or international attention to particular crises,^17^ changes in data collection over time introduce new biases into documentation efforts, biases that complicate efforts to conduct comparisons across time. This challenge is not unique to our topic. As Hendrix and Saleyhan write about conflict event data [53, p. 404], particularly with the advent of digital data repositories: ‘In collecting data on say, protest, simply incorporating new sources as they become available can lead to what falsely appears to be an upward trend in the aggregate data and underreporting on countries that are less digitally connected’. The same holds true for our data, whether in relation to media interest, to the discrepancies of geographical focus and digital connectivity, or to the growing role of social media as a source.

Nevertheless, publicly-available data remain crucial in raising and maintaining awareness about this topic, and in supporting open policy discussions in ways that may not be possible using non-public or confidential data. In places where field data are not collected to triangulate or verify information, publicly-available data remain crucial to understanding the scale of the problem and potential solutions, in particular as they can be openly discussed, unrestricted by confidentiality concerns. The datasets discussed in this study illustrate how database parameters shape the data collected using publicly-available sources, which, in turn, are often identified using processes that are not always fully transparent and algorithms and curatorial practices that reflect hidden biases [54]. In short, no data source can capture everything [5], but understanding the scale and type of underreporting in available public data is important in the quest to document and more importantly, to reduce attacks and their impact.

Addressing underreporting and documenting the full scale of attacks on health care will require multiple efforts to increase data sharing and collaboration across a range of actors, systems, and activities [see also 5]. This includes the development of data sharing mechanisms for non-public information, thereby allowing for comparison and reconciliation of event data across databases. Collaboration with on-the-ground health networks—international, national, and especially local health providers and medical professionals—is key to improving information data quality and access. Additional systematic work is likewise required to (1) translate definitions into effective online search mechanisms to better capture the full range of events, and (2), increase the diversity of languages and local sources to better capture events across geographies. Moreover, to increase the potential for capture-recapture methods in documenting attacks, it is important to note source information and to adopt a consistent approach to checking sources, in order to address potential concerns about the quality of data and the reliability of sources [29, 53]. These actions will advance efforts toward more systematic documentation of attacks on health care, address underreporting, and ideally, toward the mitigation of their effects on health care providers and the populations affected by war and violence.

This article represents a step in this direction by increasing transparency about the processes of collecting public data and highlighting the multiple layers of biases that may result. At a minimum, analysing the processes of data collection and comparing these datasets has prompted us to more critically examine our sources and the processes of generating data on attacks. We hope this analysis, in turn, will advance our understanding of publicly-available data about the extent and scale of attacks on health care and their limitations, and support a wide range of policy processes towards better protection of health care from violence.

## Limitations

The study is limited in several ways. First, the study compares only 47 countries/territories that fit the WHE definition and only for one year so it cannot generalise across time or geography. Second, it examines only how the parameters of data collection (e.g., definitions and data sources) affect the composition of datasets and does not examine the public data as such, which themselves are subject to bias [28, 31, 53]. Thus, our focus here is on the processes by which we collect data and not the selection biases of the sources themselves (e.g., what news media choose to report). Third, the data do not permit a detailed comparison of the number of health care professionals and patients wounded or killed, or the type of facility, since many public source reports did not specify the exact number of victims or provided an imprecise description. Having such data points would allow a more in-depth analysis of attacks and comparison of the datasets. Finally, the study covers the collection of publicly-available data in 2017, a time before social media was widely used by citizen journalists or activists to report on individual incidents and before advancements in open source investigations.

## Data Availability

The full datasets used and analysed in the current study are available from the corresponding author. The full 2017 SHCC overview dataset from which the media reported events used in this comparison were taken is publicly available on the Insecurity Insight page of the Humanitarian Data Exchange at https://data.humdata.org/dataset/shcchealthcare-dataset.

## Footnotes

1 This campaign is no longer active, although MSF issues periodic statements related to attacks on its health care staff and facilities.

2 Other important publicly-available data sources on attacks on health care include the Physicians for Human Rights (PHR) data on Syria and Yemen, the Yemen Data Project, as well as the Syrian-American Medical Society (SAMS) data on Syria.

3 For this article, we compared only parts of the full 2017 SHCC data. In total, SHCC identifies 701 events for that year, some of which originated from other organisations or sources (e.g., confidential reports). These events were therefore excluded from this analysis.

4 As reported below, we excluded Syria data from this analysis for this reason.

5 Both the WHO Surveillance System for Attacks on Health Care (SSA) and the SiND collect information about attacks via field-based organisations. As indicated above, WHO field offices collect information via its partners on the ground and input these directly into the SSA. The SSA started in December 2017. Insecurity Insight has partnership agreements with 30 organisations that submit their incident reports for inclusion in the Aid in Danger project, which started in the early 2010s. While both organisations report these data in aggregate figures when publishing on attacks on health care, all field-based data were excluded from this analysis, since the article focuses exclusively on publicly-available data only, and not on the differences between field-based vs publicly-available information.

6 Tilly defines event catalogs as ‘﻿a set of descriptions of multiple social interactions collected from a delimited set of sources according to relatively uniform procedures.’ [44]. Event-based data are common in conflict datasets, in which individual observations of violence (in this case, attacks on health care) are separately and uniformly coded according to different categories and using standardised rules.

7 Doctor, nurse, paramedic, medic, ambulance, physician, hospital, health care worker, health care clinic, health facility, health care facility, kill, attack, threaten, execute, assassinate, bomb, shot, ambushed, road side, kidnap or abduct, hospital, hospital, medical centre, doctor, nurse, paramedic, medic, health, health care worker, health care clinic, health facility, health care facility, physician, ambulance, medical, clinic, NGO. WHO personnel on the WHO Attacks on Health Care team reviewed and compiled the data for their quarterly reports.

8 Aid Worker Security Database (AWSD); Armed Conflict Location & Event Data Project (ACLED); Council on Foreign Relations (CFR); Physicians for Human Rights (PHR); the United Nations Office for the Coordination of Humanitarian Affairs (OCHA) reports; plus documentation/press releases from Médecins Sans Frontières and the International Committee of the Red Cross.

9 These included but were not confined to major English-language news sources such as the BBC, CNN, or the New York Times, as well as humanitarian-focused news outlets such as The New Humanitarian (known as IRIN in 2017), Thomson Reuters AlertNet, UN Wire, DevEx, and ReliefWeb, and tailored security updates on insecure environments, such as Foresight Security, which included local news sources. Most of these news outlets provide curated daily news summaries sent directly to an email inbox. This constitutes an open source dataset, as per the Berkeley Protocol [47]. Notably, in 2017, open source information in the SiND rarely originated from social media. Multiple staff identified and coded these events, undergoing training from Insecurity Insight, which were cross-checked by a single staff member.

10 Note that the comparison dataset did not include the full 2017 SHCC data (701 events). Events in Syria and those originating from other organisations or sources (e.g., confidential reports) were excluded from this analysis.

11 Within the WHO SSA (post 2018), obstruction accounts for a larger number of events.

12 ACLED uses open source data to systematically document and disaggregate conflict-related events, such as violence against civilians, protests and riots, and other types of political violence [51].

13 In 2017 ACLED did not collect data in Latin America and the Caribbean, Europe, Central Asia and the Caucasus (Afghanistan excepted) or East Asia [52].

14 ACLED does not always make the link to the original source. As a result, some question whether ACLED data can be considered open source data for the events for which no source link is available.

15 While field-based data can complement publicly-available data, practical work to combine these into a single dataset highlights multiple challenges. These are often related to missing data points in field-based collected data intended to protect the identity and safety of those who shared it. This is a separate discussion needing further investigation.

16 See https://www.who.int/initiatives/eios

17 For example, violence against doctors, nurses, and other medical personnel during COVID drew attention to attacks on health, just as the US bombardment of the MSF hospital in Kunduz eventually led to the adoption of UN Security Council Resolution 2286, on the protection of the medical mission. Similarly, attacks on health are better reported in some armed conflicts and absent in others. Local media and anguage coverage can help address these discrepancies.
